# Scintigraphy for transthyretin cardiac amyloidosis diagnosis in Austria, Germany, and Switzerland from 2021 to 2024. A survey report

**DOI:** 10.1007/s00259-025-07601-8

**Published:** 2025-10-02

**Authors:** Oliver Lindner, J. Bucerius, B. Burchert, T. Derlin, R. R. Buechel

**Affiliations:** 1 Institute of Radiology, Nuclear Medicine and Molecular Imaging, Heart and Diabetes Center North Rhine-Westphalia: Herz- und Diabeteszentrum Nordrhein-Westfalen, Georgstrasse 11, Bad Oeynhausen, D-32545 Germany; 2 Department of Radiology, Division of Nuclear Medicine, Medical University of Graz, Graz, Austria; 3 Department of Nuclear Medicine, Cardiac Imaging, University Hospital Zurich, Zurich, Switzerland

**Keywords:** Cardiac amyloidosis, ATTR diagnosis, Scintigraphy, Positivity rate, International survey

## Abstract

**Purpose:**

Bone scintigraphy using technetium-99 m-labelled phosphonates is increasingly applied for imaging of cardiac transthyretin (ATTR) amyloidosis. However, there is limited data on how frequently this technique is used.

**Materials and methods:**

To address this issue, we added questions about the number of patients referred for nuclear medicine scanning for suspected cardiac amyloidosis and the proportion of positive results (Perugini score of 2 or 3) to the 2024 survey on myocardial perfusion imaging conducted jointly in Germany, Austria and Switzerland.

**Results:**

The number of participating institutions in Austria, Germany and Switzerland was 12, 170 and 16, respectively. The number of patients scanned for cardiac amyloidosis was 1487, 4029 and 824 with a positivity-rate of 41%, 37% and 49%.

**Conclusion:**

The survey data reveal an increasing diagnostic effort for cardiac amyloidosis from 2021 to 2024, with a similar trend observed in all three countries. The average positivity rate in 2024 was almost 40% vs. about 33% in 2021. The results from 2021 to 2024 across all participants demonstrate improved pre-selection by the referring physicians but also increased awareness and improved training among nuclear medicine physicians.

**Supplementary Information:**

The online version contains supplementary material available at 10.1007/s00259-025-07601-8.

## Introduction

Bone scintigraphy using technetium-99 m-labelled phosphonates is a sensitive, easy-to-perform, highly cost-efficient, and widely available method of imaging cardiac transthyretin (ATTR) amyloidosis. According to several guidelines, cardiac ATTR diagnosis can be made based on typical echocardiographic or magnetic resonance imaging (MRI) patterns and positive scintigraphy with bone-seeking radiopharmaceuticals together with the absence of monoclonal proteins in the serum and urine [1, 2]. Data from patients who underwent bone scintigraphy for oncological or rheumatological reasons revealed myocardial uptake in 0.36% of cases, which was related to age and male gender. The intensity of uptake was not specified in this study [3]. To date, there is just limited data on how frequently bone scintigraphy is used in diagnosing cardiac amyloidosis in real-world clinical routine [4].

To address this issue, we added two questions to the myocardial perfusion imaging questionnaire regarding nuclear medicine scanning in suspected cardiac amyloidosis.

## Materials and methods

For the 2024 survey, the updated database from the 2021 survey was used to contact departments and physicians practicing nuclear cardiology. In Austria and Switzerland, nuclear medicine departments were identified in collaboration with the Austrian Society of Nuclear Medicine and Molecular Imaging (OGNT, formely OGNMB) and the Swiss Society of Nuclear Medicine (SGNM/SNNMN), respectively. The survey commenced in January 2025 and was closed at the beginning of May 2025.

The participating departments were asked.

1. for the number of patients referred for nuclear medicine scanning for suspected cardiac amyloidosis and
2. the proportion of positive results (Perugini score of 2 or 3).

Further aspects on nuclear medicine amyloid diagnosis, such as monoclonal protein testing, the radiopharmaceutical used or the acquisition protocol (planar imaging with or without additional SPECT) were not asked.

The results presented refer to the year 2024 and the previous survey, conducted in 2021.

## Results

**Fig. 1:**
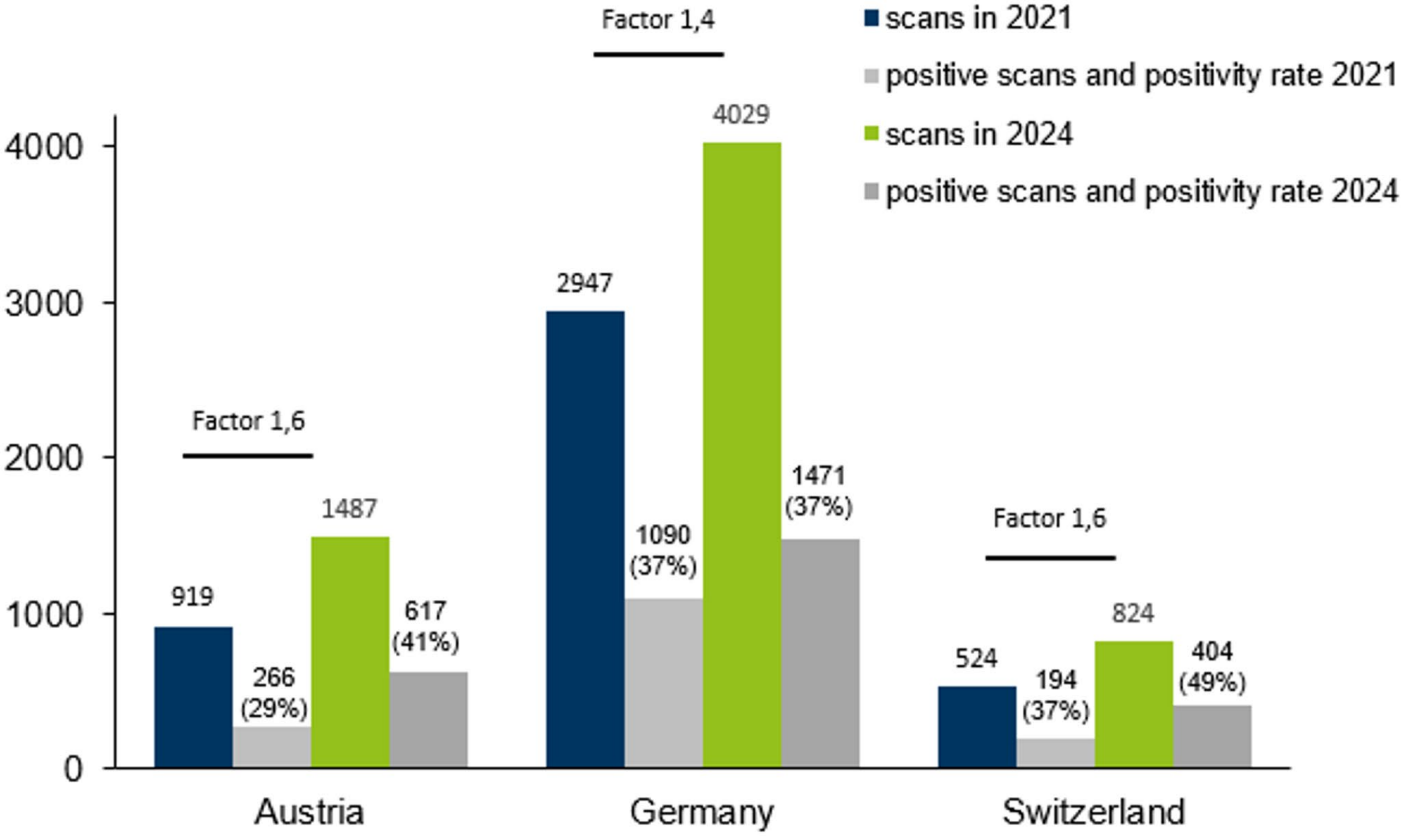
Number of bone scans for ATTR diagnosis and number of positive scans in Austria, Germany, and Switzerland from 2021 to 2024

In the 2024 survey, the number of participating institutions in Austria, Germany, and Switzerland was 12, 170 and 16, respectively. The number of patients scanned for cardiac amyloidosis was 1487, 4029 and 824 with a positivity-rate of 41%, 37% and 49%, respectively. The results are presented together with the 2021 data as shown in Fig. 1. The detailed results among the different types of institutions (private practices, hospitals, university hospitals) are given in Table 1.

**Table 1:** Institutions by country in nuclear medicine ATTR diagnosis and respective positivity rate in 2024

	Austria (*N* = 1487)			Germany (*N* = 4029)			Switzerland (*N* = 824)	
	PP	HO	UH	PP	HO	UH	HO	UH
ATTR diagnosis (%)	11 (164)	44 (654)	46 (669)	50 (2015)	33 (1330)	17 (684)	38 (313)	62 (511)
Positivity Rate (%)	16 (26)	35 (288)	54 (25)	32 (645)	40 (532)	45 (308)	56 (461)	45 (230)

*PP* private practices, *HO* hospitals, *UH* university hospitals

Absolute numbers are set in brackets

The number of institutions in Austria, Germany, and Switzerland which provided data in both queries was 9, 119, and 10, respectively. They showed a positivity rate in 2021 of 30%, 41%, and 54% in 2021 and of 46%, 38%, and 47%, respectively in 2024.

## Discussion

The findings of the current survey highlight an increasing diagnostic effort for cardiac amyloidosis from 2021 to 2024, with a similar trend observed in all three countries. The average positivity rate in 2024 was almost 40% (vs. about 33% in 2021), with growing rates observed in Austria and Switzerland during the study period over all institutions.

Considering the subgroup of departments which participated in the 2021 and 2024 survey their positivity rates reflect the trends observed in Austria and Germany, whereas in Switzerland a mild decline was seen.

The results across all participants demonstrate improved pre-selection by the referring physicians but also increased awareness and improved training among nuclear medicine physicians.

False-positive results, e.g., due to residual blood pool activity, are unlikely to confound the positivity rate as only Perugini 2 and 3 results were considered.

In Austria and Germany, university hospitals exhibited the highest positive rates, a finding that can likely be attributed to the presence of associated amyloidosis centers and a pre-selection of patients with high suspicion of ATTR. Contrarily, the relatively low positivity-rate in private practices is probably due to a less specific diagnostic approach, primarily aimed at ruling out cardiac ATTR.

Of course, the survey data is incomplete. However, the evaluation of previous surveys with official data from the National Association of Statutory Health Insurance.

Physicians in Germany and the Austrian National Public Health Institute has shown that a satisfactory level of representativeness was achieved in Germany and Austria [5]. Unfortunately, there is no official data available from Switzerland. However, based on the number of respondents, it is reasonable to assume that the sample is similarly representative to that in Germany and Austria.

## Conclusion

The results of nuclear medicine imaging for cardiac amyloidosis in the three European countries Austria, Germany, and Switzerland in 2021 and 2024 reveal an increasing trend in diagnosis. This is certainly due to growing awareness, improved training, more therapeutic options, and an ageing population. Consequences include a greater demand for specialised care and ultimately higher healthcare costs. The next surveys will reveal whether the observed trend is going to continue.

## Supplementary Information

Below is the link to the electronic supplementary material.

Supplementary Material 1

## Data Availability

The data on which this article is based are not publicly available in order to protect the privacy of the departments submitting their data. This was explicitly promised to all participants. Data can be passed on anonymously on reasonable request.
