# Supplementary material for: Scintigraphy for transthyretin cardiac amyloidosis diagnosis in Austria, Germany, and Switzerland from 2021 to 2024. A survey report
Source: Eur J Nucl Med Mol Imaging. 2025 Oct 2;53(2):1113–6. doi: 10.1007/s00259-025-07601-8 (PMC12830428; doi:10.1007/s00259-025-07601-8)
Supplement: Supplementary file 1 — Supplementary Material 1 [file 259_2025_7601_MOESM1_ESM.pdf]

Umfrage der DGN/ÖGNT/SGNM
zur Myokard-SPECT für das Jahr 2024

Wenn Sie keine Myokard-SPECT und Amyloidosediagnostik durchführen, bitte bei 1.1 eine 0 (Null) eintragen.

1.1 Anzahl **Patienten mit** Zuweisung zur Myokard-SPECT 2024	Patienten
1.2 Anzahl der **Stress**-Myokard-SPECT (ergometrisch oder medikamentös 381)	Untersuchungen
1.3 Anzahl der **Ruhe**-Myokard-SPECT 2024<br>(Hierzu zählen auch die Ruhe-SPECTs, die vor oder nach der Stress-SPECT durchgeführt wurden.)	Untersuchungen

1.4.1 **Stress**-Myokard-SPECT mit ergometrischer Belastung	Patienten
1.4.2 **Stress**-Myokard-SPECT mit Adenosin	Patienten
1.4.3 **Stress**-Myokard-SPECT mit Regadenoson	Patienten
1.4.4 **Stress**-Myokard-SPECT mit Dobutamin	Patienten

2.1 Anzahl Patienten mit 1-Tages-Protokoll (Stress und Ruhe-SPECT an einem Tag)	Patienten
2.2 Wie viel % der Stress-SPECT wurden als gated-SPECT durchgeführt?	%
2.3 Wie viel % der Ruhe-SPECT wurden als gated-SPECT durchgeführt?	%
2.4 Wie viel % der Myokard-SPECT erfolgten mit Schwächungskorrektur?	%
2.7 Verwendeten Sie 2024 Scores bei der Befundung?<br>☐ nie (< 10 %) ☐ selten (10 – 50 %) ☐ häufig (50 – 90 %) ☐ immer (> 90 %)	
2.8 Mit welchem Gerät wurde 2024 die Mehrzahl der Myokard-SPECT durchgeführt?<br>☐ Einkopf-SPECT ☐ Mehrkopf-SPECT (ohne CT) ☐ SPECT-CT ☐ CZT-Kamera	

2.9 Haben Sie während des Generatorengpasses im Herbst Myokard-SPECTs mit Tl-201 durchgeführt? ☐ nein ☐ ja, bei Patienten

3.1 Anteil Zuweisungen zur Myokard-SPECT 2024 von Hausärzten	%
3.2 … von niedergelassenen Kardiologen	%
3.3 … von sonstigen niedergelassenen Ärzten	%
3.4 … aus dem stationären Bereich	%
3.5 Haben sich seit der letzten Umfrage 2021 Änderungen bei den Zuweisungen ergeben?<br>☐ nein ☐ mehr Zuweisungen ☐ nicht bekannt<br>☐ weniger Zuweisungen durch: ☐ Stress-Echo ☐ kardiales CT ☐ kardiales MRT ☐ Koronarangiographie	

4.1 Anzahl Patienten zur Amyloidosediagnostik mittels Skelettszintigraphie?	Patienten
4.2 Wie hoch schätzen Sie dabei den Anteil positiver Befunde (Perugini 2 oder 3)?	%
